# Clinical Illustrations of the Diseases of India, &c.

**Published:** 1846-10-01

**Authors:** 


I. Clinical Illustrations of the Diseases of India, as exhibited in the Medical History of a Body of European Soldiers in a series of years from their Arrival in that Country. By *William Geddes*, M.D. &c. 8vo. pp. 492. Smith, Elder, and Co. London, 1846.

II. Remarks on the Dysentery and Hepatitis of India. By *E. A. Parkes*, M.B., late Assistant Surgeon of H. M. 84th Regiment. 8vo. pp. 271. Longman and Co. London, 1846.

During the last eighteen months we have had the opportunity, on more occasions than one, of directing the attention of our readers to the subject of tropical diseases, and of describing and illustrating some of them at considerable length, in our reviews of the recent works of Webb, MacGregor, and Wilson. When we think of the numbers of young medical men, whom their fate or good fortune is continually sending to our East Indian and West Indian possessions, and at the same time reflect upon the (too often) conflicting opinions that have hitherto prevailed, and still prevail, as to the management of some of the most common and destructive diseases of these climates, not another word need surely be said in the way of argument for the extended analysis which we propose to make of the two new volumes, whose titles stand at the head of this article. Both are the productions of able and experienced men, who faithfully describe what they have seen, and religiously avoid all unnecessary speculations.

Dr. Geddes served as Surgeon of the Madras European Regiment for four years, from the month of May 1829 to the same date in 1833. Almost all the facts, observations, and conclusions contained in the volume now before us, have been derived from this quadrennial period of service. The regiment consisted of nearly 700 men; more than one-half of them were of Irish extraction; the rest were chiefly English, with not more than 42 Scotchmen. The age of the recruits, when they joined the regiment, was usually from 20 to 23 years. Little more than one-fifth of the entire number had been, the year before Dr. G. was appointed surgeon, above three years in India; so that the great bulk of the regiment consisted of young lads. It was at first stationed at Masulipatam, on the sea-coast of Bengal, and remained there until the following December, when it marched for the inland station of Kamptee, near Nagpore, where it arrived in the beginning of March, 1830. It continued there up to the close of our author's service. Dr. Geddes gives some interesting details respecting the geographical position, and so forth, of the two stations, as well as respecting the meteorological characters of the successive seasons, while he was with the troops; these will be read with chief interest by the Indian practitioner. We must not, however, pass over what he says about the food of the soldiers; as this point of hygiene seems to us to have a most important bearing on the amount and severity of the prevailing diseases, and the results of their therapeutic treatment. Let us therefore carefully note the particulars with which Dr. G. has furnished us in the following passage:—

"The food of these European troops may be described as being substantial and abundant. It differs from that made use of by the same class in their native country, chiefly in the greater consumption of animal food, which generally forms a part of every meal throughout the day. In the preparation, also, of their victuals, there is a much greater employment of the capsicum and of various aromatics,—particularly in the curry, which is an invariable accompaniment to every dinner,—than in the colder climate; and the frequent use of the preparation just mentioned, with other reasons, leads to the article of rice becoming a prominent portion of almost every meal. Acidulous liquids, as sour coagulated milk, butter-milk, tamarinds, or lime-juice, and the like, are also abundantly employed.

"The subject of drink forms an important one for consideration in the life of an Indian soldier. A system has been in force in that country (the Madras presidency and the year 1833, are here particularly alluded to) based, apparently, upon an idea of the necessity of the use of ardent spirits by the European troops, whereby a certain portion is allotted daily to each recruit from the period of his arrival in India, the price of which is deducted from his pay. Many circumstances lead to the recruits becoming thus accustomed to such a stimulus; and there are few individuals, accordingly, who omit during the remainder of their service, to swallow their daily allowance of arrack: a spirituous liquor, in strength, as supplied to the troops, little inferior to brandy: and of this two measures, of forty to the gallon, form the daily appropriation to each European soldier."* P. 15.

The enormous quantity of 10,000 gallons of arrack was consumed in the space of twelve months! But, as if this were not sufficient, the men

---

* Mr. Parkes says upon this point:—

"The diet of European soldiers in India, varying necessarily at different places, is as a general rule far too rich and stimulating; hot curries, carelessly made by native cooks, are used several times every week for dinner; and vegetables in many places are scarce, or of indifferent quality. Soldiers often refer the origin of their complaint at once to their diet, and to my own knowledge many men have supplied the place of the curries by rations purchased out of their own scanty funds. It often happens that an European regiment, quartered with one or two companies of English artillery, will show a much greater per centage of sickness; the habits of both corps are the same, with one exception: artillerymen, being in small bodies, are easily looked after by their officers, and they are generally more careful about their diet. Again, married men, who are not in a mess, are always more exempt from both dysentery and hepatitis than single men. If this is not attributable to their food being better cooked, the circumstance is inexplicable. It is an extraordinary thing, that out of one hundred and fifty married men in the 84th regiment, only two died during a tropical service of thirty months, while in the same period the mortality among the single men was above nine per cent. The two deaths referred to were from phthisis and from delirium tremens. Some influence may be given to the habits of married men being more regular than those of single men, but in a small station, where little debauchery goes on, the influence cannot be great."

"A supervision of the whole system of diet among European troops,—not as regards commissariat supplies, which are generally excellent, but as respects the cooking of these, and the time of meals, the encouragement of teetotal societies by every allowable means, and the formation of day and night guards, differently clothed to prevent the effects of the great daily thermometrical range of some Indian stations,—are measures which would, I am convinced, at once reduce the list of duodenal hepatitis, and would probably even diminish the number of cases of dysenteric, febrile, and primary hepatitis." P. 228-9.

are allowed to have more by paying for it: "the canteen is kept open for the sale of liquors to the men; and it has been ascertained, that the average annual expenditure of arrack and European spirits, wine or beer, as sold in that place, amounted to 1440 gallons of arrack, 56 dozen of brandy, or gin, 18 dozen of wine, and 60 dozen of beer, during the period referred to in the above Table. The average number of individuals present with the regiment, throughout this interval, was about 500. The quantity of intoxicating liquors, thus used by the men, would be considered great, if equally divided amongst them, and taken at divided intervals; but, in point of fact, while some soldiers observe great regularity in never exceeding their ration allowance, and a few restrict themselves to a less quantity, or even abstain from the use of arrack altogether for limited periods, generally under the sanctity of an oath; others, again, lose no opportunity of becoming intoxicated, and often retain themselves, more or less, in this condition, for several days."

The smoking of tobacco, in the form of cigars, was very general; and some men, it is believed, were addicted to the use of Opium, or to that of *bang*—the highly narcotic leaf and flower of the *Cannabis Sativa.*

After detailing a variety of minute particulars respecting the amount, and so forth, of sickness during each year that he was attached to the corps, Dr. Geddes gives us the following summary of the general results:

"From the foregoing details, it appears that an average strength of 533 European soldiers, afforded an annual average of 1,332 admissions to hospital for a space of five years; or a monthly average of 111 cases of disease. This exhibits a degree of sickness equal to 2½ admissions to each soldier within the year; and their stay in hospital appearing, on an average of the whole, to have been 12¼ days, the mean loss to the service from confinement there, throughout the above period, was at the annual rate of about 31 days, or, in other words, one month in twelve. Again, on an examination of the observations from which these averages are taken, it appears, with regard to the number of times each individual was in hospital during the five years alluded to, that these varied from once to 45 times. Of those men who were present at head-quarters during the whole of this period, four persons only had not been in hospital within this time; and of these, also, two had been unwell, but from their situation as staff-sergeants, they had not been placed on the sick-list." P. 75.

With respect to the mortality during the period of service alluded to, we are told that—

"The number of deaths included in the above tables of disease, as having occurred in hospital from 1st July, 1828, to 1st July, 1833, amounts to 133; being one in 57 of the sick under treatment, or somewhat less than two per cent. To these, however, ought properly to be added seven individuals, who either died a natural death before being brought to hospital, or whose diseases—contracted while at head-quarters—proved fatal, when the persons affected had been sent to a distance for the benefit of their health. The whole, being 140, forms the mortality from natural causes, in the course of five years, in a body of Europeans in India, averaging 533 individuals; this approximates to 26 per cent. in this period, or an average of five per cent. of the numerical strength in each year. Deaths from Cholera, however, being removed, the rate of mortality is reduced to 20 per cent.: or an annual average of four per cent." P. 81.

The prevailing diseases were Fever, Hepatitis and its consequences, Abdominal and Cephalic Inflammation, Rheumatism, Dysentery, Diarrhœa, Cholera, Indigestion, and Syphilis. One-fourth of the entire number of cases under treatment consisted of Fever ; and there was a like proportion of "local complaints." Dysenteric disorders range next in point of frequency, being about one in every eleven. Then follow Rheumatism and Indigestion. To these succeed (in the order mentioned) Syphilis, Cephalic Inflammation, Diarrhœa, Hepatic Disease, Abdominal Inflammation, and lastly Cholera: the cases of this last disease did not exceed $\frac{1}{74}$th of the whole number. We may, *en passant*, remark that there is no such thing, however frequently it may be named in common parlance, as what is usually called a "seasoning fever." The disease, that the recruit may first experience after his arrival in the East Indies, depends on a variety of circumstances not immediately connected with the mere change of climate; such as the nature of the locality to which he is sent, his habits, exposure to weather, and so forth.

We commence with the consideration of

### Fevers.

This order of diseases is at once the most frequent and most important of all that are met with in our East Indian Hospitals, as well of the native as of the European troops. During the space of five years, nearly 1800 cases are reported to have occurred in the Madras European regiment. They are arranged by Dr. G. under the heads of Continued, Ephemeral, Intermittent, and Remittent Fevers. The two first form but a small proportion of the entire number; it is with the two last that we shall have chiefly to do with in the following observations : we shall therefore begin with them.

### *Intermittent and Remittent Fevers.*

With respect to the complete identity in nature of these two most frequent forms of pyrexial disease, our author remarks :

"It is observed that, on the arrival of a body of men at an unhealthy station, the hospital becomes gradually filled by patients who are found to have attacks of fever daily, or every second day. In some of them, the frequency of pulse and other symptoms descend to a natural state between each paroxysm of fever; and in others, a certain degree of pyrexia remains at the accession of the following paroxysm. Patients are daily received under each of these circumstances; and in cases of relapse, the intermittent of one admission has occasionally become a remittent in the next, and *vice versâ*. The succession of symptoms and progress of each paroxysm, correspond in either variety; and at the same time it is found that the remedy which is specific in one form, possesses the same powers in checking the other. In short, apparently the produce of the same cause, showing similar symptoms, and removed by the same means, there seems no difference between these two forms of disease, but that of severity. It is true that, in certain stages of some instances of remittent, symptoms become superadded which have no corresponding appearance in cases of intermittent; but they are the result of the violence, or, along with this, the duration of the disease; and their frequent occurrence, or otherwise, indicates the degree of severity in an epidemic attack, or, occasionally, the method of treatment employed. Believing, therefore, all these cases of a fever in paroxysms to be of the same nature, whether having a remission, or an intermission between them, it is intended to apply the term *Paroxysmal* to these diseases in general; in contradistinction to that of *Continued,* as given to Synocha, and others." P. 90.

These fevers were comparatively rare in the regiment while it remained at Masulipatam on the sea coast, compared with what was the case at Kamptee, commencing with the first rainy season there. They prevailed more in the months of August, September and October (during and immediately after the rainy season) than in any other part of the year; gradually diminishing, in point of frequency, during the cold months, and again increasing in March and April with the returning heat.* The prevalence of the fevers was uniformly observed to be proportionate to the amount of rain that fell during the wet season, coupled with the degree of heat of the weather before and after the fall. Much too, doubtless, depended, in respect of morbific influence, on the range or variation of temperature experienced in the course of the twenty-four hours, during the sickly season. Indeed, as Dr. Geddes observes, " an alternation of temperature appears necessary to excite the phenomena of Paroxysmal Fever; and the course of this change is observed to be the occurrence of heat *after* that of cold, combined with moisture."

The age of the individual seems also to have a good deal to do with the degree of his liability to be affected with the febrific miasm. Those men at or beyond the age of twenty-five were observed to be decidedly less liable to fever than those a few years younger. " Of 193 persons who in 1830 were under the age of twenty-five, 100 were affected in the first year of their sojourn at Kamptee, and only 38 escaped altogether; while of 276 individuals, at and beyond the age of twenty-five, 99 were affected in their first season at this station, and 81 had no fever previous to the period of the author leaving the regiment."

The same thing was observed in respect of the tendency to a recurrence, as of that to the first attack, of the disease; for there was a larger number of relapses in those below, than in those at and above, 25 years of age. Among the soldiers, the Scotch suffered less than the English and still less than the Irish. Is this to be attributed to their greater sobriety?

With respect to the influence of longer or shorter residence in India on the liability to attacks of Fever, it is by no means easy, as will appear by the following extract, to arrive at any definite conclusions.

" It appears, that those having been the shortest period in that country—who were generally, however, the youngest soldiers—were most liable to be attacked with the disease; that, beyond a short sojourn there, an interval seemed to occur where individuals appeared less liable to be affected; but that the tendency rather increased afterwards, until the age of the soldier, perhaps, became an auxiliary in enabling the constitution to resist the cause of the disease. Thus of 444 individuals, whose residence in India was ascertained on the 1st of July, 1830, in 132, whose stay there had not extended beyond two years at that date, 108 had Fever in the three following years, instead of 95; which should have been the number attacked, according to the general average. In 54, whose residence had been from two to three years, this average was observed; those from three to four

---

* September was, on the whole, the most sickly, and February the least sickly, months in the year. Such was the case at Kamptee; but this statement does not hold good as respects other parts of India.

years, who amounted to 160, had two less than the general average; 37, again, whose residence extended to different periods from four to eight years, rather exceeded the average; while of the remaining 61 persons, whose stay in India had been more than eight years, 35 only had Fever, instead of 44, which should have been the number according to the proportion at which the Fever generally prevailed. Length of residence has, perhaps, little effect in the prevention of an early attack; a fully equal proportion of the older residents being affected in the first season of their sojourn at Kamptee as of those who had been a shorter period in India. Of 22 individuals, however, who suffered from eight to twelve attacks of the disease, four had been only one year in India at the period of the first accession of Fever; five had been two years; seven had been three; and three had been four years in India. The remaining three had been seven and nine years in that country. It would appear, therefore, that the greater proportion of those having many relapses had been but a short period in India; but the effect here may be divided between the influence of youth and that of the short sojourn in a warm climate." P. 112.

Before alluding to some of the more remarkable phenomena of Intermittent and Remittent Fevers, Dr. Geddes makes the general observation that the descriptions, that have been given of these fevers by many preceding writers, would have been much truer to nature, and therefore more useful to the young practitioner, if they had been considered and described, not as distinct maladies, but merely as different degrees of one and the same disease, exhibiting the same general features, and requiring the same general principles of treatment.

With the view of establishing a distinction between the Remittent and the Intermittent forms of Fever, it has too often been the practice of authors to select only the most strongly-marked cases for description, omitting the less decided, but perhaps more important, forms of the disease; and, all the while, but little information is given as to the relative frequency with which it makes its appearance in these decided forms.

Whenever the pulse fell down, in the intervals between the pyrexial paroxysms, to 72, the case was considered by Dr. Geddes as one of Intermittent fever; and when it kept above this mark, it was set down as one of Remittent.

After a variety of minute statistical details respecting the frequency of each of the usual symptoms of Fever, we come to the following notice of the Spleen, as one of the viscera that is very often affected:

"In 35 cases, some degree of pain in the site of the spleen is reported to have been felt, at certain periods of their progress. This pain has generally been increased by a long inspiration; and, sometimes, is stated to have been so by coughing, vomiting, walking quickly, or by lying on either the right or left side. It has, occasionally, extended towards the breast and left shoulder, or towards the pubes or umbilicus. The period of the case at which this symptom has been noticed, has most frequently been about the second or third day after admission; but in some instances it has preceded this event, and in others, been of later occurrence. It has generally been of speedy removal; but sometimes has continued for a short while after the tendency to Fever has become checked. In only one of these cases was there appearance of enlargement of the spleen; and, from the effect of remedies, it seems most likely that the pain reported in the site of this organ, or near to it, has, in the greater number of instances, depended upon obstruction of the bowels near to this situation. The spleen was found enlarged in another case of Fever, after the patient had suffered twelve paroxysms of Tertian Fever, before coming to hospital; but in no other instance was any affection of this viscus to be observed." P. 130.

In all cases, without exception, the abdomen should be most attentively examined, more especially in both hypochondria, and along the head and tract of the colon. It is uniformly of first-rate importance to relieve local irritation, at the same time that we seek to arrest the constitutional disturbance. In some cases, the Headache and Vertigo are the most annoying symptoms present. Under such circumstances, the effects of the quinine require to be narrowly watched; as this medicine is unquestionably liable in some constitutions to aggravate, if not to excite, the head-distress.

As to the hour of the day at which the attacks of paroxysmal fevers usually take place, we read:—

"It has been remarked that where the paroxysms of Fever are disposed to recur at certain intervals, their accessions more frequently take place at one period of the day than at another. This fact is exhibited in the Table given in the next page, where the usual hours of attack in the cases of each type of Fever are arranged. It will be there observed, that the general tendency of the Tertian interval—as shown in the one paroxysm of the single, and in the corresponding or more perfect fit of the double cases—was to have the accession of Fever at an earlier period of the day than in the Quotidians. Thus of 421 of the latter, the attacks occurred before noon in 161, and in 260, after that hour: while of 776 single and double Tertians, the paroxysms took place in 659 in the earlier half of the day, and in only 117 in the latter portion. Another circumstance which has been remarked, is, that where there have been more than one or two admissions with Fever in the same person, the after attacks observe a considerable degree of similarity in their hours of accession as well as in their type: that, in short, where the system has become accustomed to Fever, a certain regularity of type and hour of accession is assumed: the exceptions to this rule being produced, it is believed, by accidental causes, at or previous to the commencement of the attack. It is difficult to exhibit, in a tabular form, the facts which tend to prove the truth of this observation; but it may be remarked, that they con-consist of a general view of the respective hours at which the several relapses of each patient took place; and the types of Fever which they presented. The result of this inquiry is, that, in cases where there were more than two attacks, there was generally a great predominance of the relapses of the same type, and occurring at the same period of the day; and that this was particularly remarkable among the Tertians, and their usual hours of accession; namely, the earlier portion of the day." P. 137.

With very few exceptions, the febrile paroxysms occurred at some period of the *day*-time.

The Intermittents were, upon the whole, one-fifth more numerous than the Remittents; but the relative frequency of the two forms was found to vary a good deal in different years and months. The Remittents were most numerous from July to November, and especially in the month of September. It was observed that the proportion of Remittents gradually diminished, as the residence of the men was prolonged at Kamptee. As a general result, it would seem that, when Fevers are most prevalent, they are usually the most severe, and most disposed to assume a continued type; and that they diminish in violence according to the length of time of a person's exposure to the febrific cause, and also according to the number of relapses which he has experienced. Hence, Intermittents predominate during the less sickly months, while Remittents are most frequent during those that are most unhealthy; and hence also the fact that by far the greatest number of relapses exhibit a simple tertian character. There were only thirteen instances of Quartan observed out of the whole number of the cases that were treated; these occurred, with two exceptions, in the period when least fever prevailed, viz. in the seven months commencing from October. In two individuals no other type of fever was observed; but in the remaining cases, the patients had all suffered previously either from Quotidian or Tertian fever. In four cases, the disease was checked before the third paroxysm occurred; in six cases, the paroxysms reported to have taken place were three, four, or five; in the remaining three cases, the disease had recurred at each quartan period for the space of one or two months. The cases of Quartan were observed chiefly in men about 29 or 30 years of age, and who had been from five to ten years in India.

The *diagnosis* of Paroxysmal Fever is usually abundantly obvious, if the symptoms be accurately recorded at two distant periods of the day, and due attention be paid to the nature and history of any attendant local affection. Cases, however, are every now and then occurring which will somewhat perplex the less experienced practitioner.

"There are three classes of disease, for which it is liable to be mistaken; one, where a febrile state is the predominating complaint; another, where any of the common topical accompaniments of Paroxysmal Fever exist, and are attended by pyrexia; and the third, where a Fever is present, putting on the paroxysmal form, but having its origin in some organic visceral disorder. In the first class are comprehended those ephemeral attacks arising from excitement by heat, drinking, and the like, or from disorders of the chylopoietic organs, or exposure to cold and moisture; in the second, are comprised certain cases of cephalic or splenitic Inflammation or Rheumatism, and Cholera, all of which, when Paroxysmal Fevers are numerous, may put on so much the appearance of these Fevers as to be confounded with them; and, in the third, are included cases of Hectic Fever arising from Hepatic abscess, Phthisis, or other extensive organic lesions producing constitutional irritation. In distinguishing such disorders from Remittent or Intermittent Fevers, the medical attendant will be chiefly guided by the prominence or presence of peculiar symptoms, or their more or less continued nature and duration; and it may be here remarked, as has been already adverted to, that nothing will be found to assist him more in arriving at a correct knowledge of the nature of the disease under treatment, than accurate and minute records of the symptoms of the case, combined with the information which a series of such reports will afford, respecting the previous history of the patient." P. 150.

The results of the treatment pursued in the 1210* cases which occurred in the quadrennial period of Dr. Geddes' service must indeed have been highly gratifying to him; only one proved fatal. This great success was doubtless owing partly to the promptitude with which the disease was encountered, partly to the general mildness of its character, and in part also to the skill which our author, from his long and multiplied experience,

* It would seem that nearly 200 cases more had occurred during the period referred to; but, as these cases were attended by other medical officers doing duty at the time with the corps, they are not taken into consideration at present.

had acquired in treating the fevers of India. He tells us how much less successful he had been on some former occasions, when he was in medical charge of native battalions at Seringapatam in 1823, and at Cuddapah in 1826. In the latter instance, out of 955 cases, 20 proved fatal. We must not forget to mention that, at the period alluded to, Quinine had only been lately introduced from Europe, and even the supplies of Cinchona powder were often insufficient to meet the demands. Thus, in one-half of these 955 cases, no bark or quinine was used at all. There is so much varied and curious information in the following table*—one of several dozens which our author has given in the course of these "Illustrations"—that we have much pleasure in submitting it to our readers as an example of the great pains which Dr. Geddes has expended upon the composition of his elaborate work.

Of the 20 deaths, 17 occurred among the southern Hindoos, and 3 among the Bengalees; while in none of the Mahomedans did the disease prove fatal. This was most probably owing in part to the difference of food and greater strength of constitution among the latter, and in part also to the circumstance that they reported themselves more readily sick, and gave a more intelligible statement of their complaints than the poor superstitious Hindoos. The men who suffered were chiefly young recruits; there being but three whose ages amounted to 24 and 25, while thirteen were only 18 and 20.

In fourteen of the fatal cases, the patients died in a state of delirium or insensibility, supervening upon violent and repeated paroxysms of the fever. In three other individuals, death seemed to be caused by the utter prostration that succeeded to the severe febrile paroxysms, but without the occurrence of any marked cerebral symptoms. In one of the remaining three cases, the patient, who had been affected with vomiting, diarrhœa and slight delirium, sunk just before the expected accession of the cold fit, apparently from a sudden failure of the vital powers. In the remaining two cases death was occasioned, in one by dysentery, and in the other by hectic fever, induced by abscess of the chest. So much for the fatal cases at Cuddapah. With respect to those that occurred at Seringapatam in a battalion of native infantry, 1119 strong, from 15th April 1823, to the 10th March 1824, Dr. Geddes is not able to give a very minute report; but the following are the leading facts. Out of 1503 cases admitted with fever into the hospital, 22 proved fatal.

"In these cases affection of the head was not so prominent as in those above stated. Three individuals only died in delirium or stupor; while another who had suffered from the former affection, eventually sunk by the occurrence of a sloughing ulcer on the loins. In two persons, death took place from the violence of the Fever—one of the cases being attended with cough and much expectoration; five patients died by the supervention of severe Dysentery while in hospital with Fever; and one by the accompaniment of a peculiar disease (*Beri-beri*) characterised by the lower limbs becoming paralytic, and this ending in dropsy, particularly affecting the cavities of the chest and pericardium. In 10 cases the death was occasioned by the result of a chronic state of Fever, or of frequent relapses, in which either a tendency to œdema, or a loose state of the bowels occurred; and frequently both of these symptoms became combined together. By the increase of these disorders, accompanied occasionally by returns of Fever, the patient sunk in an extremely debilitated and emaciated condition." P. 161.

* TABLE exhibiting the Prevalence of FEVER, its Types, Chief Means of Cure, &c., in a Native Corps in 1826-7.

This Battalion consisted on the 1st May 1827, of, Mussulmans, 244; Bengalees, 153; Southern-Hindoos, Gentoos, 274, and Malabars, 182; Native Portuguese, 6; Indo-Britons, 6; Total, 865.

In the solitary fatal case, which occurred in the Madras European regiment, the patient died in a state of delirium and stupor after some severe accessions of fever; there had been diarrhœa and occasional vomiting. On dissection, the liver was found large, and friable; *the stomach was filled with a liquid like coffee-grounds;* and a few superficial ulcers were observed on the mucous surface of the bowels.

An occasional very troublesome consequence of protracted fever in India is the formation of a peculiar form of *Ulcer*, that is thus described by our author.

"They commenced from a trivial sore, such as that of a blister, the friction of a shoe, or the like; and assuming a round form, with raised, thickened, abrupt, or callous edges, and an irregular, unhealthy, and dirty surface, they increased in size with considerable rapidity and much pain. The pus adhered to the surface, and the discharge was a watery or bloody ichor. After extending, occasionally by ulceration, and at other times by sphacelation, the surface began to assume a more equal and clean appearance; the edges became reduced; and granulations forming from the bottom of the ulcer the sore gradually healed. These ulcers occurred at different periods from November to April; and chiefly in men whose health had been broken by frequent returns of Fever, or by long confinement to hospital, from these or other diseases, or by a severe affection of the mouth from mercury.*" P. 156.

With respect to the subject of *treatment* of Remittent and Intermittent Fevers, all we propose to do at present is, to select a few simple memoranda from our author's narrative that may be useful in refreshing the memory of the reader.

Dr. Geddes remarks that, when he first went out to India, the usual practice in Remittent Fever consisted in the exhibition of Calomel pushed to salivation. "Bloodletting and other remedies were also employed; but without reference to the stage of the disease at which they were applied." The results of such practice were often most injurious. It is not often that this much abused medicine, Mercury, is carried to such an extent in the present day.

In the cold stage, a dose of Opium—one grain combined with eight grains of the *pulvis antimonialis*—was usually given. When vomiting, purging, or other alvine irregularity was present, a dose of Calomel—from 5 to 20 grains—was associated with the opium. In the hot stage, the great object should be to moderate the violence and shorten the duration of the febrile action. Bloodletting was not often required for this purpose; it was practised in 16 cases only. Local bleeding, by leeches—applied most frequently to the temples, in other cases to the nape of the neck, to the chest or abdomen—was extensively used, and, for the most part, during this stage of the disease. Antimonials were given in frequently-repeated doses.

"Opium, if not previously exhibited, was given, at this period of the disease, under the same circumstances as in the cold stage; and was combined with calomel, or the antimonial powder, according to the state of the stomach or

---

* This subject was considered at some length in our last Number, in the review of Dr. Wilson's Medical Notes upon China.

bowels at the time of its being used. This remedy was occasionally contra-indicated by severe affection of the head, or a plethoric, robust habit of body of the patient, and severe Fever; but, in general—as soothing the patient's feelings, checking various symptoms, and tending to lessen, and thereby shorten, the cold as well as the hot stage of the disease, while it rendered the succeeding portion of it more complete—opium was considered a valuable adjunct in the treatment of many of these cases of Fever; and was generally given where the patient was seen in either of the two first stages of the disease." P. 173.

The period at which the exhibition of a purgative, when necessary, was found most serviceable is upon the accession of the sweating stage, so that the operation of the medicine might go along with the decline of the febrile symptoms.* It is to be remembered that purgatives are not required in all cases, and are therefore not to be given indiscriminately.† We cannot quite agree with our author in the propriety of the following suggestion; would not the remedy recommended be much more advantageous in the first or cold stage?

"When the patient was first seen at this (the sweating) stage, or decline, of the disease, and the stomach or bowels were not prominently affected, an emetic of Pulvis Ipecacuanhæ frequently took the place of a purgative at this period; particularly when the admission took place in the evening. The effect of this medicine was to remove indigestible or irritating matter from the stomach, and occasionally to act slightly on the bowels; while its operation also assisted the process of diaphoresis going on at the time. An emetic was thus administered in 495 cases." P. 175.

As we have already mentioned, the treatment of the Paroxysmal fever of the East Indies is infinitely more easy and more successful since Quinine has been introduced into practice. Its use should, as an almost invariable rule, be commenced whenever the febrile paroxysm has fairly began to decline, and the skin has become moist. We are not to wait until the pulse falls, nor until any inflammatory and dysenteric, or other affections

---

* Dr. Sutherland, whose little work on "Mercury in Fevers, &c.," we were obliged, from want of space, to notice so briefly in our number for last April, remarks upon this subject:—

"There is, perhaps, no form of fever wherein it is of greater importance to be particular in the time chosen for the administration of calomel or any other remedy. The dose of calomel that would prove highly beneficial, along with a purgative extract, in cleansing the bowels of mal-secretions, if given when half the period of remission is elapsed, would have no purgative effect at all if given in the early stage of an exacerbation; and the same dose given half an hour previous to the expected exacerbation would most probably induce it, and render it more severe, that otherwise the patient might have escaped. Untimely purgatives are often the cause of renewed paroxysms."

† On the subject of those black tarry evacuations so frequently observed in cases of protracted Remittent Fever, Dr. Parkes says that, judging from the results of necroscopic examinations, the liver will generally be found much enlarged and congested, while the mucous surface of the bowels is but little affected. "The black stools probably came from the minute structure of the liver, and passed at once into the common duct without entering the gall-bladder. The black, coffee-grounds-like matter, which I have seen vomited in dysentery, comes evidently from the dark striated vessels occupying the bottom of sloughing ulcers in the colon."

that may happen to be present, be removed; for its use is by no means incompatible with that of depletory and other such remedies as may be required to relieve these complaints. By preventing the tendency to the recurrence of the febrile paroxysm, the quinine serves to facilitate and promote very materially the removal of any accidental morbid phenomena that are complicated with the primary disease.* The usual dose of the quinine was three grains, given every two or three hours. From a scruple to a drachm and a half was required to effect a cure.† The average stay in the hospital was about six days. Nearly three-fourths of the patients, 883, were discharged by the sixth day; 226 by the eighth; and the remaining 88 were there for a longer time. The quinine seemed to produce Vertigo and Tinnitus Aurium in some cases; in two or three instances these symptoms were followed by a certain degree of deafness. Occasionally its use in the fluid form was followed by diarrhœa and intestinal uneasiness.

*Continued and Ephemeral Fevers.*—We have not much to say upon this head.

"Continued Fever," Dr. Geddes observes, "in its form of a contagious disorder, as usually seen in Great Britain, has not been met with in the course of the author's experience in the East. It is true that, in certain instances of violent remittent, the symptoms, as have been above described, occasionally put on a typhoid appearance; and, in some cases afterwards to be alluded to, the disease has occasionally assumed the chief features of a Continued Fever; but in neither case was there any cause to believe in the presence of infection; while the history of the attack in the one instance, and the presence of certain symptoms in the other, showed that the febrile disorder was not originally of a continued nature, or if so, it was not a purely idiopathic disease." P. 195.

Not a few of the cases of Ephemeral or apparently Continued Fever were owing to the effects of intoxication, or of direct exposure to the sun's rays. In some instances, erysipelas or a furuncular eruption appeared upon the subsidence of the febrile symptoms. What is usually called a *stroke of the sun* (coup-de-soleil) is an attack of ephemeral or continued fever, accompanied with symptoms of cerebral congestion, which, it is well known, may terminate in stupor and death. Three fatal cases of this description occurred in our author's practice. In the more fortunate cases, a paralytic weakness of one side or of one extremity was sometimes left behind. Venæsection and purgatives were generally required. The abuse of spirituous liquors will occasion the same sort of apoplectiform attacks, which exposure to the intense rays of the sun is apt to produce.

---

* Dr. Parkes remarks that "the combination of Calomel and Quinine appears decidedly useful in Remittent Fever, in reducing the bulk of the liver and spleen." He says that the combination of the quinine with the mercurial has appeared, on several occasions, to accelerate the occurrence of ptyalism; and that, when this effect had ceased, it was re-induced by the use of quinine.

† Nine pounds, four ounces and a half in all were used in the treatment of 1200 cases. It would appear, however, that the Cinchona in powder was given at the same time; for we read, in one part, that this in powder "was generally employed, from motives of economy, in the last two days of the patient's stay in hospital, when the force of the disease had been fully checked by the Quinine."

The Second Chapter is devoted to the subject of *Diseases of the Head.*

*Cephalic Inflammation.*—During the four years of our author's service, 38 cases, to which this appellation might fairly be applied, were admitted into hospital. It is unnecessary to describe the symptoms of the affection, nor need we enlarge upon the appropriate treatment. Topical bleeding, blisters, and mercury pushed to slight salivation, are the chief remedies. If the febrile symptoms and head-affection assume anything of a paroxysmal character, Quinine must be given; but we need scarcely say that its effects require to be watched with attention, as any inflammatory action about the head is exceedingly apt to be aggravated by bark in any form.

Four cases out of the 38 proved fatal; in two, by serous effusion; in another, the serum was mixed with purulent matter in the cavity of the cranium; and in the last, there was simple inflammation or congestion of the encephalon, but without any traces of effusion.

Cephalic Inflammation sometimes terminates in Paralysis,—generally in the form of Hemiplegia—at other times in Epilepsy. Of the latter disease, 59 cases occurred in the regiment to which our author was attached during four years. These 59 cases occurred in 12 individuals. In two or three instances, the epileptic seizure was followed by a delirium, which had all the characters of that consequent upon hard drinking—a too frequent cause, by the bye, of the original malady. The remarks of our author on the use of Opium,—usually associated with Calomel or Antimonials, and after the employment of due depletory remedies—in the treatment of Epilepsy deserve notice.

"Opium was in some cases given as an anodyne, and in others as an antispasmodic. Its powers in the former capacity were chiefly required when the disease had originated in hard drinking, or was attended by symptoms resulting from that cause; as an antispasmodic, it was employed in conjunction with depletory or other measures, according to the state of the patient, and duration of the disease at the time. It was thus exhibited in twenty-one cases of those having Epileptic fits; and in eleven of these no convulsions took place after a dose of Opium had been administered. In four instances, no fit occurred after a second dose had been given; in one, they ceased to recur after the fourth dose; and in another after the eighth; while in three cases, where one dose, and in another where two had been given, the convulsions eventually ceased, without reference to the exhibition of this medicine." P. 257.

The number of cases of genuine *Delirium Tremens*—the "horrors" as it is called by the common soldiers—is distressingly frequent among our East India troops. But can we wonder at this, when we think of the disgraceful facility with which our soldiers the recan obtain the very poison, which is the "*fons et origo*" of all the mischief. It is high time for the medical officers of the army to remonstrate with those in power on this most important subject of hygiene, until a salutary change is introduced. Dr. Parkes is certainly mistaken when he asserts that too much importance has been attached to the abuse of spirituous liquors, as a producing cause of gastric and duodenal disease among our East India troops. He has himself recommended the establishment of tea-total societies among the soldiers.

Of all the chapters in Dr. Geddes' work, that on *Thoracic Inflammation* is the least praiseworthy or satisfactory. The great blemish of the whole is the entire omission of so much as a passing allusion to the use of Percussion and Auscultation in the diagnosis of thoracic diseases, more especially of that very disease whose history occupies almost the whole of the present chapter—Pleuritis, acute and chronic. In the three fatal cases, it does not seem that the existence of the pleural effusion had been clearly made out during life, except in one instance; and in that, Nature herself made an effort to evacuate the fluid, by inducing a prominent elastic swelling between the sixth and seventh ribs on the left side. Upon an incision being made into it, nearly a quart of semi-purulent matter flowed out. The patient died ten days afterwards. No dissection took place; but our author concludes that, "from a survey of the symptoms attending the disease, the case seems to present more features of a chronic inflammation of the pleura, terminating in the formation of purulent matter, which made its way to the surface, than of any other disorder likely to have occurred.

There cannot be a reasonable doubt upon the subject. In the other two cases, it seems probable that, if a correct diagnosis had been formed, recourse might have been had with advantage to *paracentesis thoracis*. In one of the cases, the heart was found pushed somewhat to the right side, by the large quantity of the effusion in the left pleura.

The Chapter on *Abdominal Inflammation* is chiefly taken up with the subject of Splenitis and inflammatory affections of the Bowels;—the different forms of Hepatitis and their consequences being considered in a separate chapter. Out of 81 cases, reported under the official appellation of abdominal inflammation, there were 28 of simple uncomplicated Splenitis. Most of these cases occurred in persons, whose health had suffered from previous attacks of Fever, Rheumatism, Dysentery or Hepatic Disease. Perhaps the second named is the most frequent precursory affection; and it deserves to be remarked that, in the progress of several of the splenitic cases, symptoms of a rheumatic character were more or less decidedly developed.

The only enteric affection, that we shall notice, is that wherein a subacute inflammation of the *Caput Coli* constitutes the predominant feature. The narrative of one of the cases reminds us of several instances which have occurred in our own practice; and, as we have reason to believe that the exact seat and kind of the existing disease is apt to be mistaken, it may be well to give our author's description, which is very true to nature:

"There appeared a combination of weakness and irritability of the large intestines generally, along with a peculiar tendency to inflammatory action about the head of the colon, or in the right iliac region. There were, accordingly, at irregular intervals, attacks of looseness of the bowels, with a feeling of soreness in the abdomen, or a pain from the movement of flatus, which was often of a severe tearing nature. The affection of the right side of the abdomen, however, was the most conspicuous part of the disease. At first this consisted of a slight pain or uneasiness about the head of the colon, which was shortly afterwards reported as being more severe; and situated to the right of and below the umbilicus, stretching up towards the spleen on pressure. This soon became diminished; but in two days afterwards, an acute pain, in fits, was reported to extend from the right groin towards the stomach, attended with vomiting: though this was relieved in some measure by a full operation on the bowels. A soreness continued next day, increased on pressure, about the upper end of the inguinal canal; and under the operation of small doses of Sulphas Magnesiæ, occasional sharp pains were felt, and described as if two gushes of water were coming to the spot in different directions, and thus exciting pain when they met. Pain was likewise excited on drinking. There had also been some irregularity in the pulse, with sharpness in its beat, for several days. Chiefly by the operation of saline purges, the local affection became alleviated; but a lax state of the bowels, with occasional griping and flatulence, continued; and although the patient was quite easy in the horizontal posture, he felt on rising up, or on a long inspiration, as if the intestines adhered together at the former site of pain. By blistering the part, however, and remedies adapted to give tone to the bowels, recovery gradually took place; but the patient's stay in hospital extended to twenty-nine days." P. 430.

On the whole, we prefer oleaginous to saline aperients in such cases. Leeches, followed by the application of hot poultices, should always precede the use of blisters.

*Hepatic Inflammation and Abscess.*—There were 268 cases registered under this head. On a general average, about one in every 23 admissions to hospital belonged to this section; and rather less than one in every 10 individuals was annually affected with inflammation of the liver, or, its result, the formation of an hepatic abscess.

Among these 268 cases of Hepatic inflammation, there occurred 21 fatal cases of Hepatic Abscess. In one-half of the cases, the patients do not appear to have had any serious disease before the formation of the abscess. Between a third and a fourth of them had suffered from Dysentery. Others had suffered from Rheumatism.

"Upon the whole," says Dr. G., "with the exception of Dysentery, and perhaps of Rheumatism, it does not appear that abscess of the liver is often the consequence of any other disorder. Dysentery, too, is a very common symptom of Hepatic abscess; and it is not improbable that, in some of the cases registered thus, the Hepatic affection may have already occurred, and proved, in such instances, a cause, and not a consequence, of the dysenteric disorder. The connection, in short, between Dysentery and Hepatic abscess, is such, that it is difficult, in certain cases, to say which is the original disease; and, although due weight has been given to the attendant circumstances in forming a diagnosis, there has been a doubt—in arranging some of the Dysenteric cases of patients, where Hepatic affection has afterwards become more decided—whether they should have been considered as idiopathic diseases, or as symptomatic of the liver disorder." P. 311.

In some cases there was but a single abscess, in others there were several. Of 29 cases, the abscess was single in 23, and multiple in 6. When the abscess was single, it was much more frequently in the right than in the left lobe. The small abscesses were generally diffused through the entire substance of the liver. With respect to the situation of the solitary abscesses, it appears that, of the 23 cases, "12 were seated in the upper part of the lobe, near to the diaphragm; and in three of these, the disease had communicated with the lungs, and been partly brought up by expectoration. In four, the site of the abscess was more deep seated, or in the posterior part of the liver. In one, it was placed near to its convex surface, and had been evacuated by an artificial opening in the right hypochondriac region; in another, it was large and solitary, in the right lobe, but extending partly to the left; and in three, the abscess was situated near the margin of the right lobe, which adhered, in two of these cases, to the colon. Of the solitary abscesses in the left lobe, one was situated near its concave surface, where it had burst into the cavity of the abdomen, and the other was placed in the upper part of the lobe."

The *symptoms* of Hepatic abscess are often exceedingly indistinct and obscure. There is no single symptom which is present in all cases, however similar these may be in the seat and size of the existing abscess; or which may not attend other cases of hepatic disorder, when no suppuration has taken place. Hence the not unfrequent errors that are committed in the diagnosis of the disease in question. Many cases are set down as examples of Dysentery, or chronic Diarrhœa; which, in others, the symptoms have been attributed to Fever, either paroxysmal or continued, Abdominal Inflammation, &c. With respect to *pain* in the right hypochondrium, as one of the symptoms, our author remarks that—

"Although frequently indicative of the locality of the disease, it has in some instances been wanting altogether, or during certain periods of the complaint; in others it has been felt at a distance from the situation of the abscess; and it has occasionally shifted its chief site during the progress of the disorder. This symptom has also varied greatly in degree, as well as in its extent; and this independently of the size of the abscess." P. 344.

Dr. Parkes makes very similar remarks. According to Dr. Geddes' experience, pain in the right shoulder was present in almost every case where the abscess was seated in the convex part of the liver; but, when it was near the under surface, this symptom was generally absent. Sometimes it continued when the hypochondrial pain ceased; at other times, it vanished, and again returned with or without the uneasiness in the side. The shoulder-pain may be felt in or about the joint, over the clavicle or scapula, or even in the upper part of the arm. One patient described it as if his arm had been dislocated.

In very many cases, there is no outward swelling or fulness to denote the existence of any purulent collection. In one case only did the abscess point externally. In four, it burst inwardly; in three of these into the substance of the lungs, and once into the cavity of the abdomen.

The character of the attendant Hectic Fever differs considerably in different cases. Sometimes it takes on all the violence of Synocha or Inflammatory fever; and at other times it is apt to be mistaken for a genuine Remittent or Intermittent. In a third set of cases, the febrile symptoms are obscure and indistinctly marked; perhaps there is nothing more than recurring rigors, followed, it may be, by slight flushes of heat. The sweatings are often very profuse; occasionally, however, they are scarcely noticed. There is, as might be supposed, considerable diversity in the state of the pulse. Whenever it is uniformly above the standard of health during the remissions of the fever, the case must be regarded as suspicious.

From what we have now said, it is obvious that, in many instances, the physician must exercise very great circumspection, and that in all he should make repeated and most pains-taking examinations, before he makes up his opinion as to the diagnosis of the malady. On the one hand, he should most carefully avoid attaching an exaggerated importance to any one symptom that may be present, and, on the other, he has to guard himself against his suspicion being lulled by the absence of those symptoms that he might naturally expect to find. Much harm, it is obvious, must be done by errors of diagnosis in this disease; for, assuredly, if a case of incipient or existing hepatic abscess be treated as one of uncomplicated Dysentery or of Fever, positive and very serious injury may result from the treatment that will almost inevitably be pursued. It is well known that a relaxed state of the bowels is very generally present in cases of hepatic abscess, and this symptom may be so prominent as almost completely to mask every other sign. Again, in reference to fever, it seems not improbable that the system may be under the influence of febrific malaria, at the very time that serious organic mischief is going on in the liver. Such a complication will necessarily serve to render the diagnosis obscure and perplexing; as it may then be difficult to distinguish the symptoms of Hectic fever from those of malarious Paroxysmal fever.

On the interesting pathological question as to the *Relation between Hepatic Abscess and Dysentery*, the following data—collected together by Dr. Geddes, be it remembered, without reference to the point under consideration—merit notice.

"In the large intestines, the diseased appearances were generally in proportion to the degree of dysenteric affection immediately preceding death. In 13 cases, where there was either no irregularity, or the bowels were inclined to constipation, or the evacuations, although loose, were more of a diarrhœal nature, and generally unattended with blood, little or no disease, with the exception of some contraction of the colon, was met with. In the remaining cases, ulceration, or disease, was discovered in all degrees, in proportion to the previous dysentery, and varying from one or two superficial ulcers in the head of the colon to general ulceration, with thickening of the large intestines. It may be remarked, that of 28 dissections, the large intestines were found considerably diseased in ten instances. One-half of these occurred in the cases where there were numerous abscesses in the liver; in two, the abscess was seated in the upper and posterior part of the right lobe; in one, on the margin, and in another, in the upper part of the same lobe, and in the remaining case, in the upper part of the left lobe. The circumstance most usually observed in those individuals was, that where the intestines were healthy, the abscess was found either in the upper and outer part of the right lobe, or deep-seated in the lobe." P. 363.

Besides the 31 cases* of *ascertained* Hepatic Abscess, our author gives an account of 34 cases which he regarded to be " cases of probable Abscess in the Liver," from the character of the symptoms present, and in which a recovery seemed to take place. These symptoms, we may remark, were, " pain of side affected more or less by position, pressure, or respiration; protrusion of the liver; varieties of Dysentery; pain about the right shoulder; vomiting, under various circumstances; appearances indicating either vitiation, diminution, or increase of the bilious secretion; varieties of a fever considered hectic; peculiarities of the pulse and tongue, with a disordered state of the appetite for food or drink; and, lastly, disturbed sleep."

* Dr. Geddes, in discussing this very important subject, blends the results of his earlier experience in India with those of his practice during the four years that he was surgeon of the Madras European regiment.

In many of the cases under this head, the patients had suffered from dysenteric and other disorders before the development of the symptoms of hepatic suppuration. As a matter of course, there must always be more or less uncertainty as to the correctness of the diagnosis, that has been formed under such circumstances. In some instances, the probability of the opinion being correct was much increased by certain necroscopic appearances discovered in the liver, the patients having died subsequently of another disease. A brief account is given of some such cases. We shall merely notice the *post-mortem* appearances in two or three of them.

In the case of a man, who had been affected with all the usual symptoms of Hepatic abscess for several months before his death, the following is the report of the dissection.

"The surface of the liver presented, in the upper part of the right lobe, a scar of an ulcer, without any adhesions there; and the substance immediately below it was hard and white. The same appearance was presented on the edge of the right lobe, where there was a slight adhesion to the colon; and the whole of the liver was somewhat paler and softer than what is natural." P. 369.

In another case, where the patient died of dropsy, two years after the cessation of the hepatic symptoms, we read in the report of the dissection —"the liver was found to be irregularly indurated, of a pale colour; and on the outer surface of the right lobe, there was a deep indentation, as of a scar, this penetrating the substance of the liver, for some distance, in a tendinous structure."

Similar appearances were observed in three or four other cases.*

To avoid unnecessary repetition, we may as well introduce here the general conclusions which Dr. Parkes has come to respecting the connexion between Dysentery and the formation of abscess in the liver.

"I have carefully," says he, "dissected ten cases of secondary hepatic abscess, and several others, in which the dysentery and hepatitis appeared coetaneous.

"1. As ulcers exist in every case of common dysentery, and abscess only follows in some cases, the absorption of pus cannot be the true cause of production of abscess, as stated by some writers: besides, in certain cases, the ulcers in intestines, in secondary hepatic abscess, although very numerous, are small, and are in their earliest stage.

"2. I have carefully looked out for venous inflammation, and am certain that in many cases there is no process of this kind going on.

"3. If the abscess be owing to spreading of inflammation by contact, it ought always to be situated at the point nearest to the inflamed colon; but this is by no means the case.

"The mesenteric glands are enlarged and inflamed in all cases of dysentery; but,

"4. I have never seen any suppuration of them in secondary hepatic abscess.

"5. In all the cases I have examined the duodenum was free from disease, if we except an enlargement of the solitary glands generally, and of the orifices of Brunner's glands.

---

* Allusion is made by M. Catteloup to the occasional occurrence of the cicatrices of former abscesses of the liver, in the bodies of some of the French soldiers who died in Algeria.—Vide *Medico-Chirurgical Review* for January of the present year, p. 61.

"6. So far from there being an immense secretion of vitiated bile, in many of my cases the hepatic secretion was suppressed; the gall-bladder was generally empty, or contained merely a thin red or brown fluid. Whatever influence these causes may have in certain cases, they are certainly not the general agents in the production of abscess."* P. 113.

According to this gentleman's observations, the tendency to the supervention of secondary hepatic abscess is greater when the ulcers of the colon are numerous, small, and widely disseminated, than when they are large, less frequent, and are surrounded with the signs of active inflammation.

It is a curious circumstance, noted by Dr. P., that jaundice very seldom accompanies a case of hepatic abscess; for either there remains a free exit for the bile, or else its secretion is wholly arrested. Most medical writers allege that, when the last-named occurrence takes place—the complete suspension of the biliary secretion—the patient inevitably becomes jaundiced. Not so says Dr. Parkes: "I have never seen any reason to believe that the non-secretion or the non-separation of the bile from the blood will produce jaundice. I have never seen jaundice in any case of hepatic abscess, and, in fact, in no case in which secretion has been totally arrested. I therefore, with all respect, differ from those pathologists who suppose that the liver merely separates the biliary principles from the blood, as the kidneys do urea."

It is the opinion of certain writers that Hepatic Abscesses sometimes become speedily absorbed, the purulent matter being supposed to be eliminated and carried off either by the bowels or the kidneys.

"The author's experience does not tend to strengthen these inferences. In several instances, a discharge of a purulent appearance has been observed from the bowels; but this has not been peculiar to cases wherein abscess in the liver, or in any other organ, has been suspected. In some, it has been apparently the discharge from an ulcerated state of the bowels, as the consequence of a dysenteric affection. In others, it seems to have been the result of a change in the mucous secretion of the intestines; and being occasionally attended with a state of constipation, has been quickly removed by the aid of purgatives. An appearance of purulent matter in the urine, again, has been only recorded in one case of all those included under the head of Hepatic Inflammation." P. 399.

Dr. Parkes will not admit that the purulent matter is ever carried off by the kidneys. "I have seen thick, apparently purulent, deposits in the urine, and have heard them called 'decidedly purulent;' but these are mere collections of vesical mucus, of a particular kind, and exactly similar appearances† are seen in pyelitis and catarrhal inflammation of the bladder,

---

* These observations are more especially applicable to cases of *chronic* Dysentery. With respect to the *acute* stage of the disease, Dr. P. says that, according to his experience, Hepatic abscess will be found present in every fifth or sixth patient dying at this stage of the malady.

† "I am aware that this opinion is opposed to that of many Indian surgeons who attach great importance to this so-called appearance of pus, and believe they can distinguish it by the eye from the vesical mucus, resulting perhaps from reaction of acrid or altered urine. (Conwell and others). One thing I must protest against, viz. the opinion that pain and prominence of the right side, subsiding after such an appearance in the urine, is a proof that abscess existed and has been cured. Such an opinion is a complete begging of the question."

where there is no suspicion of pus being formed any where. These deposits are soluble with effervescence in acetic and nitric acids. No coagulation was ever observed from heat or nitric acid."

This gentleman considers that "the question whether abscess, once formed in the liver, ever becomes cured by the absorption of the pus, must be considered still undecided." He evidently inclines to the negative side of the question.

Hepatic Abscess is comparatively rare among the natives of India. On this point, Mr. Parkes makes the following remarks :—

"An interesting question arises, as to the infrequency of hepatic abscess among the dark nations of India, compared to the common occurrence of this condition in Europeans resident in the same parts. In Asiatics, equally important changes take place in the large intestines in dysentery, and in the liver and spleen in malignant and remittent fever, as in Europeans. I have been fortunate enough to have had opportunities of observing this both in Bengalees of different castes and nations, and in Hindoos, from the south and west of the Peninsula, and in Burmans. Why, in hepatic diseases, so wide a difference should occur, is a circumstance that may hereafter throw much light on the pathology of liver diseases. There are many peculiarities in the diseases of Asiatics which will some time or other amply repay research. I have noticed that in phthisis the process of softening seems delayed. Phthisis is not uncommon among Hindoos; and I have seen several cases in which it was not accompanied by cough and expectoration, but simply by debility and diarrhœa. In such instances the tubercle in the lung assumes the form of hard grey masses or nodules of various sizes, without yellow matter or appearance of softening." P. 249.

We have no intention to enter upon the subject of the *treatment* of Hepatitis and Hepatic Abscess. The only point, that we wish to draw the reader's attention to at present, is the very important—and, we much fear, not well understood—one as to the exhibition of Mercury, when there is reason to believe that an abscess is forming, or has already formed, in the substance of the liver.

Dr. Geddes says of the cases, where the presence of hepatic suppuration was actually ascertained after death, that "the system was attempted to be brought under the action of mercury; but, although in one or two of the earlier stages of the more chronic cases, some degree of salivation was excited, in general no effect was produced by the calomel, beyond a slight ulceration of the gums."

This statement is in accordance with what has been said by Annesley, and other experienced writers: vide *Medico-Chirurgical Review,* for Oct. 1845, p. 503. Dr. Parkes, however, entirely denies its truth, and says that "mercury, when properly administered, produces salivation as rapidly in Hepatic Abscess, as in any other inflammatory disease." The exemption from salivation in some cases, he attributes to constitutional peculiarities in the individuals. There seems very little doubt but that the system may become mercurialised, when suppuration is going on; but it is equally true that the existence of this morbid process exerts a strong counteracting influence to the full action of the mineral. But, be this as it may, the important point for the practical physician is to determine if this powerful remedy should ever be given with the intention of affecting the system, when there are good grounds to believe that suppuration of the liver has actually taken place. We regret to say that neither of our authors gives any very satisfactory information upon this question. In our review of Dr. Budd on the Liver, already alluded to, we expressed our opinion that Mercury should never be administered under such circumstances, except, indeed, for the relief of an occasional symptom; adding that, " it will then do no possible good, and will only serve to exhaust the patient's strength, and exasperate, by its depressing influence, the local malady." We regret to find that Dr. Geddes has not arrived at the same conclusion: at least, his practice has not been in accordance with it. On more than one occasion, he talks of exciting the absorption of the purulent matter by exhibiting small doses of mercury; but although we read, in the history of some of the cases of "probable abscess of the liver," that there was but little appearance of general improvement until the mouth had been affected with mercury, we do not feel in the slightest degree disposed to retract or in any way modify the opinion which we have expressed—feeling strongly assured that a vast deal of mischief is annually done, among our East India population, by the excessive and often indiscriminate use of mercury in large and repeated doses, not only in Hepatic suppuration, but also in many other diseases. Dr. Parkes has, we are glad to find, adopted nearly the same views; for he says :—

" Although salivation can be thus established, it appears to me to have a very bad effect on the suppurating liver. It often increases the purging; or if, as sometimes happens, the blood in the stools disappears after its supervention, the rapidity of increase of the abscess appears augmented. It is generally a difficult thing to discover the real effect, beneficial or otherwise, of a medicine; but I think no difference of opinion can exist on this point, that mercurial action, during hepatic suppuration, does no good, and appears to do harm." P. 246.

It must be confessed, however, that Dr. P., in other passages of his work, seems to sanction the use of mercury in cases of hepatic abscess. And then what says Dr. Sutherland?

" In the medical treatment of hepatic abscess, the use of mercurials is indicated until such period of the disease as the abscess shows a tendency to point, or in which rupture of it appears to be close at hand, or actually to have taken place. The employment of mercurials, when this state exists, must only hasten the fatal termination of the case, and preclude the very distant chance of life that the subject of hepatic abscess possesses through the processes of nature. In the earlier conditions of abscess, it is through the action of mercurials chiefly that the process of absorption necessary to effect a cure is to be set up; but after continuing the attempt by mercurials, and no appearance of the effect being produced, a prolonged persistence in mercurials would materially lessen the patient's chance of recovery. The case must then be left to nature, and assisted by nutritive diet." P. 92.

We are the more surprised at this opinion, as Dr. S. is anything but friendly to the free use of Mercury in some tropical diseases, to which it has been considered by most writers as especially applicable. One of these is that which we now proceed to examine, viz.

*Dysentery.*—As this disease is not treated of by Dr. Geddes, it is chiefly to Dr. Parkes' work that we shall refer in the following remarks. The pathological characters of the disease are thus very faithfully given by this gentleman :—

"Admitting the inflammatory nature of dysentery, the peculiarity about it seems to be, that ulceration of the large intestines occurs with great rapidity, and except in one rare form, a case never presents true dysenteric symptoms without ulceration being present. It is evidently not from the severity of the inflammation that ulceration is so rapidly and so constantly produced, for it occurs in the comparatively slight cases, as is proved by the opportunities we sometimes have of examining these, after sudden death by cholera or coup-de-soleil: the proofs of inflammation, apart from ulceration, are often only just visible on post-mortem examination. Moreover, the same amount of inflammation exists every day in the stomach and duodenum without being followed by ulceration; and, to view it in another aspect, it is hardly conceivable that inflammatory action, in cases of dysentery, so severe as to produce almost universal ulceration of from one to four feet of large intestine, could exist without co-ordinate constitutional disturbance. If the same amount (in point of space) of inflammation and ulceration occurred in the skin (the analogue and prototype of the mucous membranes), the pyrexia would be extreme. And yet cases of very severe but nonfebrile dysentery are constantly witnessed." P. 3.

The cause of the ulcerative process supervening so rapidly upon the inflammatory one is ascribed "to the glands of the mucous membrane being particularly implicated :" these glands are most numerous in the cæcum, the ascending colon, and the sigmoid flexure, and less so in the transverse and descending colon. Our author describes, with great minuteness, the anatomical changes discoverable in these glands in the different stages of the disease. We can notice but one passage :—

"Two important anatomical varieties of dysentery, are those in which ulcerations are found chiefly in the cæcum, and those in which they are principally confined to the rectum. These varieties are also generally the result of local irritants, and produce peculiar symptoms.

"Thus when the cæcum is the part affected the tenesmus is often absent or slight, the stools sometimes are partially fæculent, but there is great pain on pressure over the cæcum, and a very perceptible fulness in that region, arising either from arrested fæces or secretions, or from spreading of inflammation to the other coats and surrounding parts, producing inflammatory swelling and œdema, or in a later stage from absolute thickening of the coats of the cæcum from effusion of lymph. If the inflammation runs high, certain effects ensue, viz. ulceration of the ilio-colic valve, and the intussusception and strangulation of some parts of ilium in the cæcum. I have seen the commencement of this state of things, but have never witnessed that extreme ulceration which is mentioned by Mr. Twining.

"The anatomical variety of ulceration solely or principally in the rectum also has its peculiar symptoms; here there is generally intense tenesmus, and the stools are often nearly pure dark blood, and if the case is neglected some portion of the mucous membrane speedily sloughs, and protrudes from the anus.

"The mesenteric glands are always enlarged in dysentery, and are sometimes acutely inflamed. I have never seen them suppurated." P. 14.

That the Liver suffers more or less seriously in every case of Dysentery is proved by the well-attested fact, that the bile is invariably altered in its qualities. In many cases, this secretion is all but suspended; no bile passes by the stools, and yet none is absorbed into the system. Sometimes it is found to be thin and not viscid, transparent, of a brownish red colour, and having numerous crystalline-like particles suspended in it. At other times, the gall-bladder is full of a dark green, thick, and very viscid bile —so stringy, that it may be drawn out in filaments three or four feet in length.

With respect to the gastro-enteric membrane, it is declared to be, in most cases of Dysentery, " healthy ; in all cases of simple dysentery the alterations in the canal are circumscribed by the ilio-colic valve. In scorbutic dysentery the ilium is affected, as is mentioned under the head of that complication. Gastro-enteritis is at certain times an accompaniment of dysentery, and is a dangerous complication, on account of the obstinate exhausting vomiting which may attend it. There are various shades of vivid, dark, or hæmorrhagic redness in small intestines, particularly ilium ; enlargement and occasional ulceration in a small degree of Peyer's and of the solitary glands, most usually of the latter.*"

Dr. Parkes says that, in dysentery, the urine in general very rapidly decomposes, and acquires an ammoniacal smell. As we have already alluded to the relations between Dysentery and Hepatitis, it is unnecessary to say more upon the subject now. Our author agrees with Annesley and other writers of note in admitting that the two diseases may stand, the one to the other, either as cause or effect ; for there is one form of " dysentery supervening to disease of the liver," and there is another, " when the affection of the liver supervenes to dysentery." It is often exceedingly difficult to determine which is the primary, and which is the secondary, affection. Some writers have been too much in the habit of asserting that Dysentery is generally, or at least often, caused by a vitiated and acrid state of the bile. Dr. Parkes objects to this doctrine for the following very satisfactory reasons :—

" First.—Because, when this deranged secretion does occur, we have bilious diarrhœa, and not true dysentery.

" Second.—Because there is often no irritation of the mucous membrane of the small intestines, to which we should suppose the so-called irritating hepatic secretion to be at least as hurtful.

" Third.—Because, under this supposition, the dysentery should be present during the whole course of the disease, and in every case.

" Fourth.—Because dysentery, in like manner, complicates some diseases of the spleen which pour out no irritating secretion.

" Fifth.—Moreover, the secretion has not been proved by chemical analysis, or any other test, to be possessed of irritating qualities ; its irritating properties have been supposed, and the supposition has been received as if it were an established fact.

" Sixth.—In some cases, in which the secretion really does appear to be irritating, viz. by producing excoriation round the anus, and scalding the patient when he goes to stool, the mucous membrane of the colon, previously ulcerated in antecedent dysentery has been found by me to be healing rapidly.

---

* " I have not mentioned the adynamic variety, because this is almost always a case complicated with remittent fever or with typhus. As I have observed in a subsequent section, among the dark races the ulcers are atonic, and may require stimulants ; but by the term 'adynamic dysentery' is meant more than this, viz., a failure of the powers of life with dysentery superadded. I have never seen this apart from fever or cholera."

"So far from irritating secretion producing the consecutive dysentery, I have been led to think that the absence of all secretion has been the cause of this disease; in other words, when hepatitis has terminated in partial suppuration, and bile is still secreted, although altered in appearance, then there is no dysentery; whereas, when from extent or peculiar situation of abscess no bile is secreted, dysentery appears to supervene. This opinion is founded on a few observations only, and I mention it here simply that its correctness may be tested by the observations of others." P. 59.

The reader will probably perceive in these remarks a certain coincidence with the views so earnestly insisted upon by Dr. Macgregor, in his late work on the diseases of the North-Western Provinces of India—vide the number of this Journal for last April.

Besides Hepatic Disease, Dysentery is often associated with Remittent or Intermittent Fever. We have so recently dwelt upon this point, in our description of the prevailing diseases among our soldiers and sailors in China, that it is not necessary to do more than merely to refer to it. We pass on to a brief notice of

*Scorbutic Dysentery.*—This is a most serious and unmanageable form of the disease. It proved very fatal to our troops at Rangoon in 1824; and is always apt to occur on board transports and other vessels, when the ventilation and food are faulty. Every now and then, it will be found in some soldiers at particular stations, although the rest of the men remain quite exempt. Probably, much depends upon the original vigour and strength of constitution, as well as upon the condition of the general health of the patient for some time previous to the attack.

"A soldier will often have a certain amount of scurvy for a short time, for which he never thinks of coming into the hospital; he is annoyed with various symptoms of dyspepsia, with rheumatic pains in the legs, perhaps with an occasional eruption in the same parts of a few purpuric spots or slight ecchymoses; the rheumatic pains are chiefly in the calves, hams, or ankles, and sometimes there is burning of the feet: there is occasional bleeding from the gums, and when these are examined they are found to be slightly swollen and of a dark red colour. The whole amount of the disease, however, is trifling, and a man will generally do his duty, and gradually recover without medical aid. If, however, from any cause an attack of remittent fever or of dysentery supervenes, then this constitutional taint at once appears in the way in which it modifies the course of these complaints." P. 123.

In the Scorbutic form of Dysentery, the ileum is much more frequently the seat of ulceration and hæmorrhage from the bowels, and perforations of their coats are of more frequent occurrence, than in the ordinary form of the disease. It deserves to be particularly noticed that the mouth is always very readily affected with mercury, whenever there is any tendency to the scorbutic diathesis.

*Treatment of Dysentery.*—Is Mr. Parkes warranted in asserting that, "in the treatment of common acute dysentery, we have an infallible guide in the appearance of the evacuations? Many other symptoms, as general pyrexia, tenderness of abdomen, heat in course of colon, tormina and tenesmus, when present, are valuable as accessary phenomena; but the absence of any or all of these is never an indication, when the stools point out an opposite course of treatment."

We much doubt whether any judicious practitioner would determine his line of treatment from the characters of the stools alone, irrespective of other symptoms; and indeed Mr. Parkes, in the very next sentence after that now quoted, admits the importance of such aid in a therapeutic point of view: for he says:—

"As long as the stools are numerous (the attack being recent and uncomplicated), bloody, sanious, dark, and copious or scanty, lymphy, shreddy, or like meat-washings; or a mixture of blood and slime with or without partial fæculence or nearly pure blood, florid, or dark, mixed with a peculiar red mucus; or fæculent, yellow, copious liquid, and stained with blood; and more particularly when with these symptoms there is pain on pressure, and great tenderness, as is the case in most instances, or heat in course of colon, depletion must be actively employed." P. 140.

In the early stage of the disease, our author strongly recommends, after venesection has been employed, the repeated and free application of leeches over the cæcum and sigmoid flexure—"three times in 24 hours, till the stools become fæculent." This practice may require to be persevered in for three or four days; after which, the daily number of leeches may generally be lessened. If there be much tenesmus, leeches to the anus give great relief. After bleeding, general and local, mild oleaginous purgatives are the most useful remedies in the early stage of dysentery.* Opium, alone or in conjunction with calomel (3-5 grains), or with blue-pill and ipecac., will generally require to be administered at the same time. Dr. Parkes is not friendly, upon the whole, to the exhibition of large doses of calomel with the view of rapidly affecting the system. That the practice often succeeds, he does not deny; but then it is to be remembered that—not to mention its very frequent failure—the subsequent effects upon the health of the patient are often injurious. It is invariably contra-indicated in the adynamic and the scorbutic forms of the disease; nor should its use ever be continued when there is reason to believe that hepatic suppuration has ensued. The therapeutic action of this potent drug appears mainly to consist in relieving congestion of the capillaries, and in causing absorption of effused lymph. Hence, in our author's opinion, "the real indication for salivation is the effusion of lymph; and consequently in chronic dysentery, and in the after stages of very severe acute dysentery, in which convalescence is so protracted as to approach chronic dysentery, ptyalism slowly produced, and carried to the point of a very gentle action on the mouth, is invaluable.†"

---

* Sulphur, given in drachm doses at bed-time, has been found by Twining and other practitioners to suit better, in some cases of chronic Dysentery, than any other aperient.

† "The preparation," says Dr. P., "which I have found most useful, is the Bichloride of Mercury—commencing with doses of one-eighth to one-sixth of a grain—in combination with the preparations of cinchona. * * * The blue-pill, or small doses of calomel, with ipecacuan, gentian and taraxacum may be substituted. Blisters, and frictions over the abdomen with a mixture of Iodine and mercurial ointment, are to be used according to circumstances."

And subsequently he remarks :—

"For my own part I have ceased to use mercury in dysentery, in any other way than as an alterative, except in chronic and long protracted and recurrent acute cases. I never aim at ptyalism, and can confidently assert that my recoveries have been greater in number, and more complete, since I in a great measure abandoned the use of mercury, than when I gave it in large quantities." P. 145.

Dr. Parkes does not seem to have used Emetics (we need scarcely say of Ipecacuan), in the early stage of acute dysentery; although they are certainly among the safest and most effectual of all remedies. He reports favourably of the nitric and nitro-muriatic acids in mild cases.*

"Injections of opium, ipecacuanha, acetate of lead, cold water, suppositories of opium and ipecacuanha, give great relief to the tenesmus. Injections of the acetate of lead in large doses, such as one drachm every four hours, are sometimes very useful.

"The warm bath relieves tenesmus, and the irritability of the bladder, which so commonly produces frequent painful and difficult micturition. Cold water injections sometimes relieve this also." P. 147.

In the *adynamic* form of Dysentery, Mr. P. says that "alum combined with catechu and camphor is the best treatment, with small and frequently-repeated doses of Dover's powder between each dose of alum. Injections of alum are very useful in that variety where the intestine is so disorganised as to tear like wet paper after death." In the *scorbutic* variety, the results of treatment are often most unsatisfactory. All depletory means must be avoided altogether, or only used with the greatest circumspection. We much doubt whether mercury in any form should be administered: Dr. Parkes advises small doses of pil. hydr. ipecacuan and nitric acid, opium in enemata, a farinaceous diet with vegetables and lemonade.†

---

* In a recent number, we suggested to East India practitioners to ascertain the state, whether it be alkaline or acid, of the alvine evacuations in such a disease as dysentery. It seems not improbable that we might thus obtain a guide, in certain cases at least, for the selection of some of our remedies.

† The following practical remarks of Dr. Fergusson (in his "Notes and Recollections," reviewed in our last number) on the allowance of fruits and vegetables in acute Dysentery deserve notice, as a contrary opinion too generally prevails.

"Because the acid and subacid fruits sometimes occasion griping when in health, these, and vegetables of every kind, are strictly prohibited. They are, however, amongst the best remedies. Nearly a hundred years ago, Sir John Pringle, one of the best physicians our armies ever possessed, proclaimed that ripe grapes were a cure for dysentery. The Portuguese and Spanish physicians, when I was in the Peninsula, went farther, and to ripe subacid fruits of every kind, added lemon-juice, with the best effects. Our own faculty, in different parts of the world, have highly lauded the mineral acids, more especially the nitric, and, in an epidemic dysentery which not very long ago afflicted Ireland, after one of our best summers, cream of tartar in large doses was found to be nearly as beneficial as mercury; in short, the acids, in every shape, but more especially in that of ripe fruits, will be found excellent remedies by all who can overcome their prejudices so far as to give them a fair trial." * * * * *

"I believe, therefore, that the free, but not immoderate, use of fruits, by assist-

Whenever there is any reason to believe that Dysentery is at all complicated with co-existing Remittent Fever, we must never forget to combine quinine with our anti-dysenteric remedies. In closing these remarks on the treatment of Dysentery, it may not be amiss to remind the young practitioner that the same line of practice is not suitable in all seasons, or to all individuals alike. One year, the disease will bear depletion much better than another; and what may suit the robust and well-nourished European will be all but life-destroying to the feeble vegetable-feeding Hindoo.

### Rheumatism.

This term is applied by Dr. Geddes to those cases, "in which pain in periosteal, ligamentous, or muscular structure, has formed the chief symptom of disease; attended generally, in the two first, with swelling and other symptoms of inflammation." During the five years from 1828 to 1833, there were between 570 and 580 admissions into the hospital from this disease. One season of the year does not seem to have been more favourable for its development than another. On the whole, fewer cases occurred during the cold than during the hot months. Out of 160 patients, the muscles and ligaments were the parts chiefly affected in 47; these structures along with the periosteum in 43; the muscles only in 23, and the ligaments only in 28; in 5, the disease was confined to the periosteum; in 8, this structure was affected along with the joints, and in 3 with that of the muscles. As might be expected, the amount of complication was usually much greater in the relapse, than in the primary, cases. The rheumatic affection was not unfrequently associated with Neuralgia in some part or another. One-half of the cases appear to have been attended with febrile re-action. This was however seldom very severe. In one-half of the febrile cases, the fever was regularly or irregularly paroxysmal —either remittent or intermittent. The number of cases of relapse was very considerable. In two-thirds of the cases, the duration of treatment was from 4 to 20 days; in the remaining third, it extended from 20 to 60 days and upwards. With respect to the causes, predisposing and exciting, of the disease, the operation of previous Syphilis appears to have been the most frequent; and this, whether mercury had been used for its cure or not. The general results of our author's observations on this head are, "that from constitutional peculiarities, the syphilitic virus gives a tendency to the occurrence of Rheumatic Inflammation; and it is probable that this disposition is increased by the exhibition of mercury, when the effects of this medicine are not so severe as to induce the patient to avoid

---

ing to keep the bowels soluble, is at all times a preservative against dysentery; and that, when the disease is present, the same use is not only harmless, but, as in the case of the dysentery in Trinidad, may furnish a most important remedy towards a cure. For, if we examine the dismal records of this scourge to our fleets and armies, we shall find that its worst ravages have been seen amongst the famished garrisons of besieged towns, or in ships remote from land, while navigating the tropical seas, or in barren encampments, where fruits could not be found."

exposure during the period of its being given to him. In several instances, too long confinement to hospital, or to one position, by the ulceration of a suppurated bubo, has been considered to assist greatly in bringing into action the rheumatic diathesis."

Dr. Geddes expressly says that, while recognising the frequent influence of previous syphilitic disease in the production of Rheumatism, it is not to be supposed that the cases, so considered by him, were instances of merely secondary syphilis. "The absence of any other symptom of the venereal disease, either occurring between it and the rheumatic affection, or at the same time as the latter, or subsequent to it; and the final cessation, in many instances, of the rheumatic attack without the aid of mercury," serve to shew that such was not the case. The only peculiarities of Syphilitic Rheumatism seem to be its great tendency to affect single joints, and the small ligaments, as those of the hands, feet, or of the os sacrum, and its less frequent implication of muscular parts, and of the corresponding joints on the two sides of the body. This form of rheumatism is apt to be tedious, and is perhaps more liable to relapse than any other kind of the disease.

Not a few rheumatic cases occur after attacks of Fever, Dysentery, Cholera, and Hepatic Disease, aided, it may or may not be, by the exhibition of mercury. The constitution of such patients was usually in a debilitated, and perhaps also a depraved, state. In another set of cases, the rheumatic affection commenced without any appreciable antecedent disease. The number of cases, in which the disease exhibited the train of symptoms characteristic of simple Acute Rheumatism, as the disease is seen in this country, was very small.

As another evidence that the majority of cases of rheumatic disease in the East Indies cannot be viewed as entirely similar to what we are in the habit of meeting with at home, we may point to the consequences that so frequently follow upon its attacks.

"In all classes, the frequent recurrence of the disease, or its lengthened duration upon any one occasion, renders the patient pale, emaciated, and crippled; and other disorders, in some individuals, become generated, which eventually, in a great measure, eclipse the original disease. The most common and formidable of these are, ulcers on the skin, and certain symptoms referred to hepatic disease. By either, or, as is usual, by both of these disorders combined, or succeeding each other, Rheumatism has occasionally proved fatal; and when such symptoms are in a less degree, or when the disease has been confined to frequent attacks of, or long continued rheumatic disorder, it very often is a cause of men becoming invalided, or discharged from the service, or of officers being obliged to proceed to Europe for the benefit of their health. In the course of five years, six soldiers were invalided, or discharged the service, in consequence of Rheumatism; and four of these were young men, two of whom, discharged in 1829, appear to have owed the origin of their disease to syphilis." P. 473.

The entire system falls into a cachectic condition, which is often manifested by the formation of Ulcers or various cutaneous eruptions, affections of different bones, and general debility and emaciation. In such cases, when they prove fatal, the liver is usually found more or less decidedly and extensively diseased. The characters of the *Rheumatic Ulcer* are described to be these. It is round and irritable; the edges are raised, thickened, abrupt, or everted; there is more or less redness around it; and its surface is irregular and dirty, with an adhesive matter or a sloughy substance adhering to it. The sore is usually painful, and extends by ulceration, occasionally by sloughing. The parts most frequently affected are the lower and upper extremities; occasionally, the cheek is the seat of it. The ulcer is sometimes single; at other times there are three or four in different parts of the body.

There is another form of cutaneous ulceration observed in rheumatic patients in the East: it is thus described by Dr. Geddes.

"In the remaining cases the ulcers were more numerous, and diffused over the skin, than in those just mentioned; and, at the same time, the characteristic features of all the sores were alike. The commencement of these has been observed under two forms: first, that of a small scab, from below which purulent matter has exuded, and on the separation of which, a superficial round sore has presented itself; and, secondly, as a phlegmonous, or furunculous tumour, containing more or less of a sloughy matter, on the separation of which a sore becomes formed. This ulcer, in its progress afterwards, when disposed to increase, becomes painful, and assumes an irregular bloody surface, with raised and abrupt edges; and its margin is either of a circular shape, or irregular, from any peculiar activity in certain parts of the ulceration. This goes on rapidly for a short period, assisted, occasionally, by a little sloughing; and there is, at times, a slight degree of hæmorrhage. The edge of the ulcer then begins to be less prominent and abrupt; the appearance of the sore is somewhat cleaner; and fungous granulations rise in its centre, the pain becoming less. From this stage, the chief characteristic features of these ulcers present themselves. These are owing to the irregularities which take place in the healing process. Granulation, cicatrization, and ulceration, are seen going on in the same sore; and a very common appearance presents itself in consequence: namely, a mass of granulations, generally spongy, and raised above the level of the surrounding skin; or a cicatrized spot in the centre, with a ring around of ulceration, deep, and with edges more or less raised and abrupt, in proportion to the violence with which the ulcerating process is going on. Sometimes, again, the sore is cicatrized at one side, and is extending by ulceration on the other. While some ulcers present this irregular appearance, in other situations they are observed, at the same time, to be altogether in an ulcerating state. It is, also, no unusual occurrence to witness places which had been entirely cicatrized, becoming painful: a small round sore is formed, and this soon occupies the whole of the new skin. Ulcers are accordingly seen upon the patient in all stages; and he often becomes detained in hospital for a long period: partly from the difficulty with which the healing process is thoroughly established, and partly from the tendency there is to a recurrence of the ulceration when the sore had become, in a great measure, or entirely cicatrized." P. 482.

These ulcers were most frequently observed about the legs or thighs; sometimes on the arm or wrist, the back, or even the forehead. In some of the cases, there was a lienteric diarrhœa present. A syphilitic taint existed in a few of the patients.

The *Treatment of Rheumatic disease* varies, as a matter of course, according to not only the severity of the symptoms, but also the nature of the constitutional complication. It would seem that, out of upwards of 300 cases treated by Dr. Geddes, in 17 only was it deemed necessary to have recourse to general bloodletting. He omits to say any thing respecting the state of the blood drawn. In one-third of the cases, mercury, carried to the extent of affecting the mouth, was the remedy chiefly trusted to. It was usually associated either with antiphlogistic remedies, or with opium, antimonials, colchicum or quinine. The severity of the rheumatic affection very generally subsided as soon as the symptoms of mercurial action became evident. The average stay in the hospital of those cases, which gave way to this treatment, was about 18 days. The usual dose of Calomel was from one to five grains, two, three, or four times a day; combined with antimony, when the pyrexia was high; and with quinine, where this shewed a paroxysmal character, or where a degree of frequency of pulse existed after the other symptoms of fever had disappeared.

"In those cases where tedious ulcers occurred, and did not give way to local applications, a change was observed quickly to take place on the exhibition of mercury; but this was frequently of a temporary nature only, and, even under its continued use, the sores occasionally reverted to their original appearance; while in some instances, on its being discontinued, they began to heal. In cases of single ulcers, the curative process was frequently excited on the mouth becoming affected." P. 485.

Colchicum does not appear to have been found very successful in the practice of Dr. Geddes: it has been suspected that some of its preparations become inactive in a tropical country. Neither is Sarsaparilla mentioned with much praise. It is curious that no notice is made of the Hydriodate of potash having ever been used. This is the more strange, as so large a proportion of the cases seems to have been of that very class in which the remedy would have been prescribed in this country. Change of climate is, in many instances, absolutely necessary for the restoration of the health.

In quitting the subject of Rheumatism as described by our author, we may remark that it is abundantly obvious that this disease in the East Indies is much more frequently connected with an unhealthy or cachectic state of the system, than it is with us at home. It is not nearly so inflammatory in its character, and will not bear the same active depletory practice. It would have been more satisfactory, had Dr. Geddes informed us of the state of the blood when that was drawn, and of that of the urine in all cases. We are strongly impressed with the idea that the use of alkaline salts, in union with colchicum and hyosciamus or small doses of opium, would be well suited to a large proportion of the cases described by our author. Quinine might, or might not, be advantageously given at the same time, according to the character of the existing fever, the strength of the patient's constitution, and so forth.

After the ample analysis which we have given of the works before us, it is scarcely necessary to say a word in praise of their merits. The present volume,—for we are promised a second—of Dr. Geddes reflects the highest credit upon him for indefatigable industry and professional zeal. We strongly recommend every medical man going to the East Indies to have a copy of it at his side, as affording an excellent pattern for him to follow in the accumulation and arrangement of his observations, when engaged in practice. Dr. Geddes has done for the symptoms of the diseases which he describes, what Louis has done for the necroscopic phenomena of fever, phthisis, &c. With what success we may leave our readers to determine. The work of Dr. Parkes is one of narrower scope, but it is equally meritorious as far as it goes. It is obvious that the author has turned all his opportunities of observation to the very best account, and that he has neglected no means of illustrating the history of Dysentery and Hepatitis, two of the most important diseases which engage the attention of the East India practitioner.

